# Open innovation as a strategy for collaboration-based business model innovation: The moderating effect among multigenerational entrepreneurs

**DOI:** 10.1371/journal.pone.0265025

**Published:** 2022-06-09

**Authors:** Wutthiya A. Srisathan, Chavis Ketkaew, Wuttiwat Jitjak, Sirinthip Ngiwphrom, Phaninee Naruetharadhol

**Affiliations:** 1 International College, Khon Kaen University, Khon Kaen, Thailand; 2 Center for Sustainable, Innovation and Society, International College, Khon Kaen University, Khon Kaen, Thailand; Universiti Pertahanan Nasional Malaysia, MALAYSIA

## Abstract

The aim of this study was to investigate the existence of collaboration-based business model innovation through an open innovation strategy among multigenerational-cohort SMEs in the context of the Thailand setting. This current research identified four key antecedents of open innovation based on resource and capability review. Open innovation is examined in two main strategies: (1) open innovation breadth and depth, and (2) open innovation cooperation. Using survey data from family-owned SMEs in Thailand, we estimate multigroup structural invariance models considering four generational cohorts by age: Generation Z, Generation Y, Generation X, and Baby Boomers. The empirical results indicated that family business owners are more likely to pay attention to innovative human capital and strategic agility among Generation Y and Baby Boomers. Meanwhile, Generation Z, Generation Y, and Baby Boomers tend to understand the importance of strategic agility before they strategize their breadth and depth of open innovation. To execute an open innovation strategy, Generation Z, Generation Y, and Generation X tend to implement a partner-search strategy and then do a cooperation plan. Our findings imply that business practitioners should understand the moderating role of generational cohorts due to their experience age. There are differences among Generation Z, Generation Y, and Generation X when participating in collaboration-based business model innovation using an open innovation strategy.

## 1. Introduction

Open innovation plays an important role in enhancing the innovation competitiveness of Thai small and medium-sized enterprises (SMEs). Open innovation as a cooperation plan is generally defined as the implementation and use of internal and external ideas from a variety of partners with diverse backgrounds to offer commercially useful innovation to the market. This is opposite to the concept of "closed innovation," which is defined as the view that innovations and research and development (R&D) are developed by firms themselves. Many SMEs lack the resources to generate a large amount of in-house R&D knowledge for innovation development. Instead, what SMEs need is an open innovation model. SME’s business model innovation is a key element of open innovation processes. SMEs must be aware of and evaluate their opportunities for openness and cooperation, as well as determine their innovation capacity and potential. That’s why an open innovation strategy influences SME performance. Open innovation has been acknowledged as a key driver of collaboration-based business model performance, happening at various stages of the innovation process or persisting throughout the innovation lifecycle (both inside and between enterprises). What’s more, the ability to identify and evaluate the competitive advantages of family-business entrepreneurs’ innovative firm characteristics, such as innovation capability and network-partnered collaboration, is of key strategic importance for co-innovating among multigenerational ownership. SMEs with multigenerational ownership are confronted with several restrictions as a result of shrinking operating budgets, highlighting the need to use available resources, of which human capital is one. Furthermore, working among multigenerational ownership finds it difficult to operate. Adjustment agility is therefore required. This research undertakes an evaluation of family business performance to create a new concept, *collaboration-based business innovation*. In turn, this research identifies the factors that lead to open innovation strategy execution, assesses the effect that open innovation strategy execution has on collaboration-based family businesses, and provides the implications for SMEs and industrial policy as a whole. In this context, four central research questions are formulated. First, what are the antecedents of open innovation strategy execution? Second, are there any effects of such antecedents on open innovation strategy execution? Third, how does the relevant importance of open innovation strategy execution influence collaboration-based family business model performance? Fourth, are there any differences among general cohorts?

There has been an abundance of debate on literature as to whether or not a collaboration-based family business model performance actually exists. Del Vecchio et al. (2019) find that network membership benefits affect business processes innovation in family firms by assisting them in decoding and directing relevant flows of knowledge that improve their competitive edge [1]. Ahmad et al. (2020) find that innovation capability plays an important role in sustaining business performance, especially the owner’s family is involved in the business [2]. However, it comes to the first gap when it is still unclear how innovation capability contributes to enhanced performance. It is also required to have measurable innovation capability in a family business. Accordingly, this research takes innovation capability into account as one of the key antecedents of open innovation and reconsiders the different measures of innovation capability. Rialti et al. (2018) argue that the main reason why business improvement performance and adaptation performance are not feasible was due to the lack of agility in the firms in the issue [3]. They further find that firms that have attained operational agility are more likely to innovate or adopt more innovative process management solutions/systems [3]. When it comes to the second gap, this research brings attention back to the concept of operational adjustment agility—being able to quickly respond to and take advantage of changes by always watching and quickly improving products and services to meet consumers’ needs. Still, many SMEs may not have sufficient resources and capabilities to devote to the development and maintenance of collaborative networks as well as the creation and enforcement of intellectual property rights [4]. The human side of open innovation of family or non-family SMEs still needs more attention to human capital. Chabbouh and Boujelbene (2020) find that human capital, in terms of managerial skills and social networks, has an effect on the degree of openness and innovation [5]. However, it leads to the third gap when assessing the degree of cooperation to collaborate in innovation. It appears to be more effective for understanding collaboration-based business model performance rather than focusing only on the degree of openness to inbound open innovation practices. Thus, innovative human capital as a human resource-based antecedent of open innovation should be of interest in this current research to understand its impact on collaboration-based business model. A focus on these four internal resource and capability antecedents of open innovation is timely for family SMEs.

To address these gaps, this current research has chosen to consider open innovation strategy as an enabler between the resource-and-capability antecedents and the collaboration-based business innovation of family SMEs. The aim is to investigate if the effects of innovation capability, the evaluation process of innovation, network membership benefit, and tradition and personal skills on SMEs’ strategic open innovation execution across different levels of entrepreneurial generations to create a collaboration-based SME business model.

Using Thailand as a case study, the research builds on the cross theory between resource-based view and open innovation approach as a determinant of business innovation performance. To the best of the authors’ knowledge, this research is the first to estimate family SMEs’ collaboration-based business innovation performance while also identifying key resource and capability determinants of open innovation strategy. In addressing the three research questions posed above, this research provides a number of significant contributions to the body of knowledge. First, this current research provides holistic approach to collaboration-based business innovation performance on the theoretical foundation of resource-based view and open innovation. Second, the empirical results from structural equation modeling reveal how SMEs apply and execute open innovation for a collaboration-based SME business innovation performance. Further analyses provide evidence that there are differential effects among multigenerational ownership of family SMEs, especially in Generations X, Y, and Z.

The remainder of the paper is structured as follows. In Section 2, we provide the synthesis of the literature review and develop the selected factors included in the model. In what follows, we explain the research design and method using data obtained from 563 family-SMEs in Thailand in Section 3. The results and findings are placed with argumentative discussions and implications in section 4. Finally, we conclude the study with limitations and future directions in section 5.

## 2. Literature review

**Fig 1:**
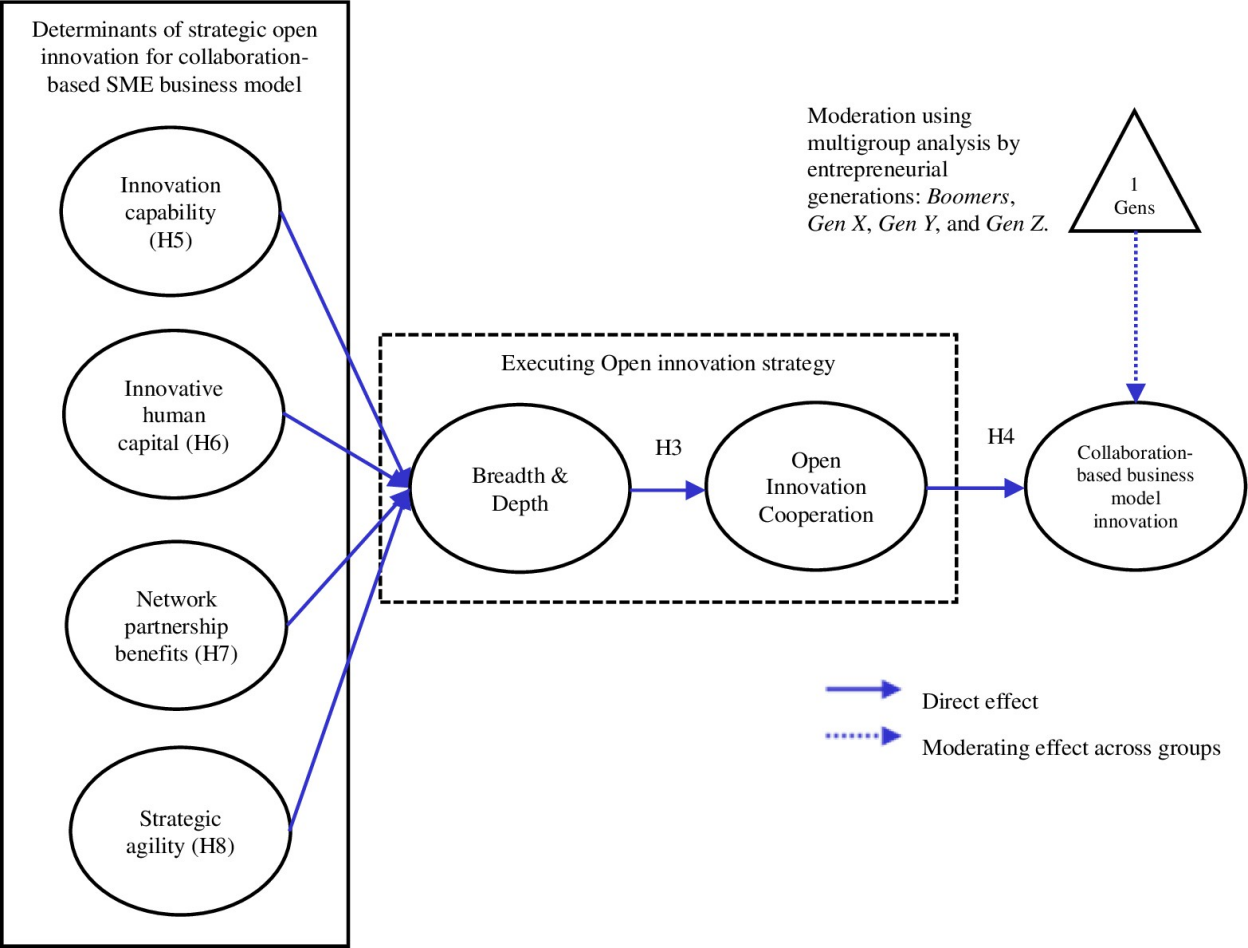
Figure generated by authors, 2022.

This section examines the significance of the research model by tracing it back to the theory on open innovation and the resource-based perspective. The first stream of the literature is focused on family-owned SMEs across generations. The second stream presents an overview of open innovation is presented and discusses why collaboration-based business innovation performance matters. The last stream ends with the identification of antecedents that affect open innovation theory and collaboration-based business innovation performance. **Fig 1** below illustrates the research model. The following subsections explain the model variables and underlying hypotheses.

### 2.1. Family-owned SMEs across generations

Family-owned SMEs are characterized by the coexistence of family and business systems, which may emerge a specific bundle of capabilities and resources [6]. In family SMEs, the family is at the heart of the operation, with family members connected by a sense of responsibility and loyalty and for the firm’s success [7]. When considering individual social networks as social capital, it is important to clarify their roles to a firm-level exploration network [8,9]. Otherwise, when multi-networks overlap, it results in multiplexity or paradox of openness, thereby causing tensions among actors in collaborative innovation [10]. There are no clear lines between the business and family systems, which makes multiplexity in family businesses a lot like living in a tight-knit community with strong norms, trust, and rules. Thus, family-owned SMEs may not be as flexible as non-family-owned SMEs [11]. Family businesses with a high degree of interdependence between family members are more likely to share information, work collectively, and offer support [6]. Thus, it can be summarized that there is a relationship between family involvement in the firm and family-to-business support, and this relationship’s effect may have on their business model innovation.

The theory of generational cohorts has made a few notable contributions to the field of business model innovation. As posed by Karl Mannheim in 1928 [12], the premise of generational cohort theory is that a generation of individuals who grow up in the same political, economic, and social environments will have a *similar* set of views, attitudes, and behaviors during the early stages of life *experience* [13,14]. Pure generational cohort theory says that a generation can be shaped by unexpected events like terrorist acts or war, and it can be shaped by new technology like digital media, which can change the way those generational cohorts think about things. Prior research by Kariv et al. (2014) finds that situations, difficulties, and resources encountered at each development stage of business model innovation will be interpreted differently among generational cohorts [15]. Thach et al. (2021) show that the events that take place in the year of their birth and the subsequent 10 to 20 years have an impact on each generational cohort [14]. We concluded that experience age plays a role in the management of business model innovation.

This current study segmented four main generational cohorts: Generation Z (between 1997 and 2009); Generation Y (between 1981 and 1996); Generation X (between 1965 and 1980); and Baby boomers (between 1946 and 1964). Please note that the year of birth relies on the arguments about generational differences suggested by Kotler et al. (2021) [16].

However, it is important build a better narrative about business that takes *societal issues* into account when it comes to its main business model. If there are leftover profits to be only addressed in the business model while viewing society as an *add-on*. This would not please either baby boomers or millennials (Generation Y) because both age cohorts want business to be a positive force in society. Both want business to work to build stronger communities, cleaner environments, and more meaningful and better jobs. It all signals that the role of generation matters. For example, in conditions in which family business owners are motivated to deeply create new business venture because the new venture creation pertains to new business unit as a result of cooperation that will directly affect them. When seeing the great benefits from open innovation cooperation, family business owners are more inclined to open up their innovation process/activities and develop new joint venture. This is how collaboration-based business model innovation takes place. The degree of open innovation cooperation to develop collaboration-based business model innovation depends on family business owners’ experience age. When they perceive that their experience age to share knowledge is lower, generational cohorts by experience age moderates the effects of cooperation on the propensity for collaboration-based business model innovation. We developed the following hypothesis:

**H1:** Relationships between open innovation cooperation and collaboration-based business model innovation will be *different* depending on generation cohorts.

They also influence what happens before an open innovation strategy is used, so the breadth and depth of openness for each generation are going to be different. Another hypothesis was proposed:

**H2:** Generational cohorts will have *different* effects on the breadth and depth of openness based on the antecedent factors of open innovation, which are made up of innovation capability, innovative human capital, network partnership benefits, and strategic agility.

### 2.2. Open innovation

The second stream of the literature is focused on the theory of open innovation. The term open innovation was first coined by Chesbrough (2003) in his book, which mentioned the new paradigm for creating and profiting from technological innovation [17]. This concept is opposite to closed innovation, which means firms believe that innovations are developed by them for their own benefit–no need for cooperation [18,19]. Many SMEs were struggling to adjust to and recover from the effects of a shortage of capabilities and resources [20]. Many resorted to open innovation, which is a collaborative approach that plays to the strengths of all key stakeholders and may offer innovative, unexpected solutions [21,22]. Open innovation is thus a type of cooperation that is worthwhile to engage in whether or not SMEs are in such short supply [23]. This comes to the reason why open innovation is required as a theoretical base.

Since 2003, the literature shows us an evolution of open innovation theory. Open innovation involves the business management model for innovation that promotes collaboration with organizations and people outside the firm [24]. This current study agreed that understanding and mastering each of the innovation evolution stages is what leads to the very best open innovation has to offer. In the early era, *Open Innovation 1*.*0* was focused on cross-functional approaches, cross-licensing, and value networks. These triple helix innovation models are developed by academia (universities), industry, and government to promote economic and social development. Following the quadruple helix model of innovation, open innovation evolved into a more customer-centric formula. Thus, in this *Open Innovation 2*.*0* era, it is centered around understanding customer segmentation, innovation ecosystem, cross-fertilization, orchestration, and value constellation. However, SMEs that share knowledge must keep in touch with a lot of partners to come up with new ideas. From a birds-eye view, those SMEs act like a swarm, looking for a win-win situation because they work well together in networks, communities, and so on. Thus, the era of *Open Innovation 3*.*0* emerged. In this way, collaborative SME clusters and networks are formed with communities that are both flexible and stable enough to store and use collective learning in multi-agent systems. We also call this era *Enterprise 3*.*0*. The rise of the trend towards automation and data exchange in manufacturing technologies and processes (i.e., industrial internet of things, etc.) changed the industrial path to interconnection. Thus, the quadruple helix model is worth keeping an eye on the fact that some of the stakeholders in innovation are now interacting with public relations, customers, and the media, while others are interacting with the environment. The open innovation notion has started to move to more digital and sustainable regimes and implement *Open Innovation 4*.*0*. This era is also given importance in *Industry 4*.*0*. The rise of Sustainable Development Goals (SDGs) has become a rather hot potato. The new attention is given to human, social, economic, and environmental sustainability. It was suddenly shifted to *Society 5*.*0*. In other words, *Open Innovation 5*.*0* will be the university-industry-government-community-environment integration to provide social solutions. The quadruple and quintuple innovation helix frameworks are also activated. This era has great concern about how technology can empower and enhance sustainability. Thus, we take the importance of the environment and public society into account. The current research views the interactions among quadruple and quintuple innovation helix stakeholders as the knowledge base to conceptualize open innovation in terms of breadth, depth, and cooperation plan. They will be used in the next section to shape open innovation breadth and depth before influencing the open innovation cooperation plan.

#### 2.2.1. Open innovation and collaboration-based business model innovation

Open innovation in this current study is focused on (1) breadth and depth and (2) cooperation plan. The literature (see Cheng and Huizingh 2014 [25]; and Lopes and De Carvalho, 2018 [26]) shows that open innovation can be divided into its core innovation processes such as inbound, outbound, and coupled open innovation. These three core processes are based on (1) knowledge exploration and exploitation [27], (2) technology exploration and exploitation [28] and (3) external search breadth and depth [29]. However, the establishment of long-term business collaboration is important for SMEs to improve their innovation activities, capabilities, and resources, the creation of network partner breadth and depth as is required for openness collaboration [30]. This is because the breadth and depth of partners in OI collaboration regarding explorative knowledge content influences firm-innovation performance (e.g., novelty and efficiency). Zhu et al. (2019) argued that open innovation as the horizontal (i.e., breadth) and the vertical (i.e., depth) strategies positively affect firms’ new product development speed [31]. When it comes to new product development, the explicit role of business model innovation cannot be ignored. This is because business model innovation elaborates how firms interact with external partners and delineates how to manage transactions with other firms [32]. Hence, open innovation breadth and depth fits better with the novel and efficient business model [31]. Once the identification and involvement of knowledge network partners (i.e., helix stakeholders) is done in the process of open innovation breadth and depth, the open innovation as cooperation plan is discussed, agreed, and signed by those stakeholders [33]. In the end, the cooperation plan enables open innovation projects to be the most effective. The cooperation process reflects different usages of knowledge sources—as a consequence of the breadth and depth—that can be used to plan, do, measure, act, and replicate open innovation before new joint ventures are created. It comes to the reason why open innovation breadth and depth is related to open innovation cooperation. Moreover, when there is an interplay between innovation networks, the different types of value and beneficiaries, and finally resources, it is dedicated to collaboration-based business model innovation. Linking open innovation to collaboration-based business model innovation, it can be interpreted that when it comes to cooperation, such a cooperation within and across firms’ boundaries shapes both the design and operation stages of business model innovation. That is why open innovation cooperation affects business model innovation. The following hypotheses were developed:

**H3**: Open innovation breadth and depth positively affect SMEs’ propensity for cooperation.
**H4**: SMEs’ propensity for cooperation positively affects SMEs’ collaboration-based business model innovation.

### 2.3. The antecedent factors

*Innovation capability* The capacity to innovate is a predictor of firm-level innovation [34]. The previous definitions of innovation capability are featured as follows. Laforet (2011) defines innovation capability as the potential or ability to create new ideas or innovations [35], while Martínez-Román et al. (2011) define it as internal capability, especially related to the creation and appropriation of organizational knowledge [36]. Thus, this research defines innovation capability as the capability of innovating firms to integrate the firm’s core skills and resources to generate innovation. Carrasco-Carvajal & Garciá-Pérez-De-Lema (2020) pinpoint that innovation capability plays an enabler role in fostering open innovation [37]. They further illustrate that (1) innovation capabilities enable key stakeholders such as customers to interact with the innovations they develop, and (2) such a firm with knowledge-sharing capabilities would be better at sharing existing knowledge with its network partners [37]. Therefore, this research implies that innovation capability has a positive relationship with the success of collaborative innovation. Ahmad et al., 2020 argued that when family involvement in business increases, there may be an impact on the capability to innovate, thereby impacting its strategic perspective performance [2]. Innovation capability is one predicter of how firms assess their internal capabilities to open up their innovation process. This is how firms use innovation capability to improve their business innovation performance. As a result, this leads to the following hypothesis:

**H5**: Innovation capacity positively affects open innovation breadth and depth.

*Innovative human capital* McGuirk et al. (2015) divide human capital into tangible elements (e.g., education and training) and intangible elements (e.g., job satisfaction and willingness to change) [38]. It is re-titled as innovative human capital. They further addressed that these elements play an important role in fostering firm-level innovation [38]. Chabbouh and Boujelbene (2020) argued that human capital elements (e.g., strategic vision, managerial skills, social networks, and R&D capacity) appear to affect the degree of openness to innovation, further affecting firms’ innovation performance [5]. Human capital is also important when it comes to internal innovation capacity. This internal (open) innovation capability is as the cultural openness to innovation [39]. Open innovation in the sense of Lichtenthaler and Lichtenthaler (2009) is often linked to R&D personnel and the presence of technical talents inside the firm [5,40]. Through their technical and scientific skills, SMEs would be able to absorb new knowledge from external sources, attract network partners, and thus exploit new opportunities for collaboration [41,42]. Hossain and Kauranen (2016) suggest that human capital in terms of knowledge depth at individual level are necessary for open innovation in the setting of small and medium-sized enterprises (SMEs) [43]. It can be concluded that human capital is likely to influence open innovation, thereby impacting firm performance. Hence,

**H6**: Innovative human capital positively affects open innovation breadth and depth.

*Network partnership benefits* A closer look at knowledge flows in family firms’ open innovation shows one factor that family-owned businesses should be concerned about—network partnership benefits. In this research context, it can be defined as firm partners that collaborate across borders to share the core values of the network in their innovation activities. Thus, network partnerships are like external knowledge sources [9]. Naruetharadhol et al. (2020) indicate networks as an implementer to foster inbound and outbound open innovation propensities [44]. Naruetharadhol et al. (2022) further find that collaborative networks positively explain the implementation of open innovation [18]. According to resource-based theory, when one network member contributes more resources, all network partners benefit. Collaboration in a business confederation may thus assist family SMEs in obtaining advantages such as finding new business and/or innovation prospects, gaining access to strategic expertise, and enhancing the entrepreneur’s professional profile [1,45]. In a value network, possible cooperation modalities include strategic alliances, R&D collaborations, and joining a network of other value networks [46]. Networks as a result of partnership collaboration may provide significant benefits to family businesses by assisting them in decoding and overcoming flows of limited source of internal knowledge, thus enhancing their competitive edge. Accordingly, we imply that there may be a possibility that the benefits arising from network partnership may positively influence open innovation in family firms. We assumed that:

**H7**: Network partnership benefits positively affect open innovation breadth and depth.

*Strategic agility* The term *agile* first came up in the business and innovation literature when it was used to describe flexible manufacturing systems (FMS) [47]. Agility and flexibility are often used together; the relationship between flexibility and agility is similar to the relationship between competence and capability [48]. Agility is a capacity that is outwardly oriented, while flexibility is a capability that is inwardly focused, and is thus an antecedent of agility [49]. The capacity to explore and exploit market arbitrage possibilities is one of the most distinctive characteristics of agility [50]. Agile firms must be used to efficiently obtaining resources and harmonizing capabilities, as well as to gathering the necessary assets, knowledge, and networks in a timely manner [51]. This perspective is consistent with not only the understanding of agility as a dynamic capability [50], but also an innovation paradigm for firms, which may require a group of meta-capabilities [52]. According to Lu and Ramamurthy (2011), organizational agility encompasses two distinct concepts: market capitalizing agility and operational adjustment agility [53]. Shin et al. (2015) argued that it appears that the strategic aim of SMEs toward agility can improve operational performance and customer retention, but not financial performance [49]. We first imply that it is possible that agility may affect firm performance. Liao et al. (2019) illustrate that when seeing inbound and outbound open innovations as strategic resources, it was found that openness affected firms’ dynamic capabilities in terms of market capitalizing agility and operational adjustment agility, thereby impacting business model innovation [54]. To differ from the study of Liao et al. (2019), we consider strategic agility as a capability antecedent. This is because open innovation (culture) requires sufficient flexibility and agility in a firm’s resources so that they can be used to support the innovation projects [39,55]. Thus, more flexible and agile, more open. It comes to the next hypothesis:

**H8**: Strategic agility positively affects open innovation breadth and depth.

## 3. Research methodology

All procedures performed in studies involving human participants were in accordance with the ethical standards of the institutional and/or national research committee of Khon Kaen University, Thailand with the Ethics committee’s declaration and its later amendments or comparable ethical standards.” All participants give verbal consent to participate in this survey. The protocol was approved by the Committee on the Ethics of Human Research of Khon Kaen University (Protocol Number: *HE653009*). All surveyed data was protected under the privacy and personal identity information of all respondents.

The arguments in the literature review show that this study is part of a new field of business model innovation that is still growing. Thus, there is a tendency to use explanatory and survey research methods, which are typical of quantitative approaches, to provide insights into the setting of a problem [56]. Our aim is to find out the antecedents of collaboration-based business model innovation performance. This suggests that a more quantitative approach would be needed. This study employed a structured research process and relied on quantitative data from an online survey to uncover and analyze family SMEs’ business model innovation performance. The study also focuses on identifying factors of open innovation and causal effects between them. Deductive reasoning with positivism as a philosophical viewpoint is used in this study, where the emphasis is on measuring variables and testing hypotheses that are linked to general explanations [57]. This section of research methodology addresses the research approach to data collection, sampling, and instrument variables.

### 3.1. Data collection and sampling

Primary data was collected in the form of a structured questionnaire survey design a as a technique for data collection. This is to examine the existing processes used by the theories suggested in the literature [58]. A self-administered questionnaire was used to deliver the survey through the internet. This study leveraged the number of SMEs from the database executed by the Office of SME Promotion (OSMEP) to determine the whole population (N) of 756,344 registered SMEs. According to Krejcie and Morgan’s premise (1970), the sample size for a known population was determined at approximately 384 SMEs, with a confidence level of 95 percent that the real value is within 5% of what was measured or surveyed and a level of accuracy for the sample of 50%. [59]. However, this study depends on multigroup structural invariance. There was a multi-sample group specification for family SMEs with multigenerational ownership, suggesting that *each* group needed to have at least 100 observations to perform the multigroup modeling [60]. Thus, this study set the minimum sample size required to gather data on four-generational ownership at 400.

Most Thai SMEs are family businesses, which embed strong family values, build trust, and give back to the community [18]. Philanthropy is at the heart of many high-quality family businesses around the world. They are a good model for the communities their innovation serves and also have a long-term impact on society as a whole through the values they contribute to. Consequently, family-owned business entrepreneurs were targeted for three key reasons. First, family SMEs fitted the research need for participatory innovation projects with their networking-partner role [61]. Second, they are both niche players and industry giants. They work on a wide range of innovation projects in a variety of industries, products, and businesses [62]. They may be able to draw on a much broader range of experience and collaborative R&D settings [63]. Both reasons carry a positive signal of open innovation. Third, according to the Thailand Family Business Survey 2019, it was found that 76% of Thai family-owned businesses have the next generation working in their businesses [64]. Furthermore, 96% of family businesses were expected to grow over the next two years, while 86% planned to pass on the baton to the next generation [64]. The impacts could be summarized as the role of generations impacting business model innovation performance. Hence, family SMEs’ business model innovation performance is worth investigating.

However, this current study was carried out in strict accordance with the recommendations in the Guide for human-subjects research. The protocol was approved by the Committee on the Ethics of Human Research of Khon Kaen University (Protocol Number: *HE653009*). All surveyed data was protected under the privacy and personal identity information of all respondents.

Although the SME information is available for search in Department of Business Development (DBD) Data Warehouse, probability sampling may not be applicable. This is because there is no completed list for random sampling. Purposive random sampling is applied. With this sampling technique, the sample selection depends on geographical area and inclusion criteria as follows:

- Must be over 18 years old
- Must be family-owned business only
- SME managers, business owners, entrepreneurs, and CEOs must be present. If none of the aforementioned people are accessible, they may send the questionnaire to anybody who works in a role that includes firm-level innovation only.
- Must identify the business’s location in terms of area and province.
- SMEs must be incorporated as legal entities.

The respondents will be excluded from quantitative study if one of these criteria is absent. The pilot questionnaire was sent out to 30 family-owned SMEs. This was a purposeful random sample as we needed to ensure that we sampled those SME managers, business owners, entrepreneurs, and CEOs from a variety of generational ownership. However, the comment was that some of the questionnaire content was difficult to understand; we decided to revise that content and re-distribute the survey. Please note that those 30 pilot samples will not be included in the final analysis.

To prevent a low return rate on the questionnaire, 800 questionnaires were sent out to a specific sample of family-owned SMEs for a six-month period (May–October 2021), where 200 questionnaires were targeted for each generation. Confidentiality has been assured in an online sample volunteer consent form, and respondents were provided with no identity for return. **Table 1** lists the demographic profiles of all respondents grouped by generations.

- 111 were returned for those respondents in Generation Z (18–26 years old), accounting for 19.72%.
- 183 were returned for those respondents in Generation Y (27–41 years old), accounting for 32.5%.
- 163 were returned for those respondents in Generation Y (27–41 years old), accounting for 28.95%.
- 106 were returned for those respondents in Generation Baby Boomer (above 57 years old), accounting for 18.83%. It took almost four months to collect the data from this generation. 83 were available to fill out the questionnaire paper, and we went to meet them at their physical offices. There was a hybrid survey that happened for this generation.

**Table 1:** Sample characteristics.

Characteristics		N	Percentage
Age	Generation Z (18–26 years)	111	19.716
	Generation Y (27–41 years)	183	32.504
	Generation X (42–56 years)	163	28.952
	Generation Baby boomer (57–77 years)	106	18.828
Geographical regions	The north	87	15.453
	The northeast	99	17.584
	The west	105	18.650
	The central	124	22.025
	The east	83	14.742
	The south	65	11.545
Type of family business	Agribusiness	71	12.611
	Household industrial business	100	17.762
	Factory industry business	108	19.183
	Commercial business	81	14.387
	Construction business	99	17.584
	Financial business	33	5.861
	Service business	53	9.414
	Other	18	3.197
Firm age	0–10 years	134	23.801
	11–20 years	150	26.643
	21–30 years	194	34.458
	31–40 years	68	12.078
	More than 40 years	17	3.020
The average income per month	0–20,000 baht	18	3.197
	20,001–40,000 baht	110	19.538
	40,001–60,000 baht	212	37.655
	60,001–80,000 baht	128	22.735
	More than 80,000 baht	95	16.874

**Note**: We used 18 as the starting point for generation Z as most Thai started family businesses at this age.

Overall, 600 respondents successfully filled in the questionnaire, but only 563 respondents (111+183+163+106 = 563) were validated because data supplied by 47 respondents was unreliable as it contained missing information. The response rate was given at 93.83%. A sample of 563 is sufficient to test for validity and reliability in the step of data analysis.

### 3.2. Instrumentation and variables

A survey instrument is comprised of two sections of questions as to demographics and variables of interest. We used seven-point Likert scales of agreement category to operationalize the research variables, ranging from strongly disagree (1) to strongly agree (7). **Appendix 1** shows a list of questions for questionnaire development in this current study. However, the following variables were modified to fit the context of the research, with a partial adoption from the previous studies. Innovation capability was measured using the scale developed by Ahmad et al. (2020) [2], Liao et al. (2007) [65], and Lin (2007) [66] to ask the respondents about how family-owned SMEs are able to come up with new ideas and turn them into new or better products, services, or processes that benefit the firm. Those items included (IC1) constantly obtains new talents or resources to improve our firm’s innovation processes during the last five years, (IC2) try out new ideas or ways to innovate within our sources during the last five years., (IC3) have capabilities for R&D of new products or services at a certain level., and (IC4) increase the number of new products and services introduced to the market during the last five years. Innovative human capital was designed to capture manifestations of the underlying IHC construct by McGuirk et al. (2015) [38], Chabbouh and Boujelbene (2020) [5], and Vidotto et al. (2017) [67]. In terms of the understanding, behaviors, awareness, and development of tacit knowledge *within* individuals, the respondents were asked how family-owned SMEs embody knowledge, including (IHC1) increase in the level of skills necessary to carry out the specific job, (IHC2) encourage staff to join the training program during the last five years, and (IHC3) empower employees to be creative. Network partnership benefits were conceptualized using a variety of scale developed by Chesbrough and Appleyard (2007) [68], Lee et al. (2010) [69], and Del Vecchio et al. (2019) [1]. They were asked about how family-owned SMEs would think about the benefits arising from network partnership. The item scale included (NPB1) access to R&D partnerships and corporate collaborations, (NPB2) solve business problems, and (NPB3) create new revenue streams. For strategic agility, we used four-item scale based on Cepeda and Arias-Pérez (2019) [70], Panda (2021) [71], and Ashrafi et al. (2019) [72], asking them as to how family-owned SMEs are agile when developing new innovation or improving existing one. Those measures included (SA1) respond to and capitalize on general/disruptive changes, (SA2) effective IT-business support, (SA3) encourage quick internal adjustments whenever there is a shortage of resources (manpower, funding, etc.), and (SA4) quick decision-making in the face of market changes.

This research extracted measurement items of open innovation breadth and depth, and cooperation. Open innovation breadth and depth were focused on (OBD1) search for external knowledge sources (OBD2) identify key stakeholders (such as suppliers, customers, universities, rivals, laboratory, local community etc.), and (OBD3) select key stakeholders to partner with. Open innovation cooperation was measured using the scale by Kontinakis & Zhang (2018) [33] and Kontinakis et al. (2019) [73] and focused on (OIC1) explore relevant and feasible activities and project, (OIC2) consult local stakeholders to select what to propose, (OIC3) discuss their funding, timing and expected, (OIC4) allocate probable resources, and (OIC5) execute and manage activities, projects, and risks. This is because the contents of both constructs are different themselves although they are under the main concept of open innovation strategy execution. That is, open innovation as a cooperation plan is considered at the stage by which family SMEs promote joint innovation project strategies for cooperation and details the cooperation between two or more stakeholders [33].

Finally, the main idea we attempt to focus on is collaboration-based business model innovation performance. This is being developed to understand how collaboration-based business model innovation is likely to be managed and performed once open innovation cooperation has a positive-signal impact. As items were adopted from a mix of previous studies by Zhu et al. (2019) [31] and Kariv et al. (2014) [15], they included (CBMI1) scale up the collaboration-based business model, (CBMI2) enables demand aggregation, (CBMI3) incorporate transparency into the business model, (CBMI4) offer new solutions and combinations of products/services/knowledge, and (CBMI5) brings new collaboration stakeholders in the business model innovation. The data analysis will be discussed in more detail in the next section, which is concerned with operationalizing the variables of interest.

## 4. Data analysis and results

The statistical tests were performed using the structural equation model (SEM) to verify the validity of the data and identify potential bias from the data collected. Harman’s single factor test is used to identify the common method bias (CMB) [74]. Harman’s single factor test results, using the indicators, showed a cumulative variance of 28.943% less than the 50% criterion, which confirmed no CMB. The CMB can create a systematic covariance over the actual relationship between indicators or observable variables, which results in erroneous estimations of the magnitude and significance of the relationship between structures or latent variables.

The SEM strategy is used in the data analysis to incorporate various statistical approaches such as path analysis. Confirmation factor analysis (CFA), latent variables, and SEM approaches are used in causal modeling. The IBM SPSS statistics version 26 and IBM AMOS version 26 program were used to conduct the CFA with a maximum likelihood estimation approach. In two steps, the SEM technique tests the model’s estimation [75]. The first step looked at the external CFA model to verify how each indicator and variable interacted. The validity of Goodness of Fit (GOF), convergent validity, and discriminant validity are checked. In the second step, we analyzed the internal structure model to see if the overall structure was valid, by performing GOF examination and hypothesis analysis. In the third step, we used age as a variable, using a measurement constant analysis and dividing the samples into four groups: generations Z, X, Y, and Baby boomer. A z-test was used to examine differences between factor loading of the three groups.

### Step 1: Measurement model (CFA)

- CFA was used to confirm the constituent indicators in the measurement models of internal conformity, convergent validity, and discriminant validity, using the observed variables in the measurement model of ready components [76].
- GOF. The passed threshold levels, see **Table 2**: Goodness of Fit of the Structural Model, are consistent with the concept [76]. The values of CMIN/df (2.674), CFI (0.934), IFI (0.934) and RMSEA (0.055), and TLI (0.922) were an acceptable fit.
- Convergent validity. Cronbach’s alpha and composite reliability analysis exceeded the specified threshold of 0.70; therefore, this research is valid [76]. The data shows an excellent fit for the testing model, and the investigation is conducted to see how well the data fit the empirical study [77], through convergent and discriminant validity. The values of standard factor loading (see **Table 3**) were greater than 0.70 [76]. The composite reliability (CR) with a value greater than 0.70 showed that all model variables had high discriminative accuracy [78]. The average variance extracted (AVE) value was more than 0.50, indicating that the measurement model is a single latent construct [76].
- Discriminant validity. Discrimination has demonstrated a high link between reflecting structures and path model indicators [76]. In **Table 4**, the predicted Fornell-Larcker threshold value assures that the AVE square root (demonstrated through the estimated diagonal value) should be greater than the sum of all construct relationships (shown through the estimated off-diagonal value) [77].
- **Table 4** shows the criteria result from the Heterotrait-Monotrait correlation ratio (HTMT). If the HTMT value is less than 0.90, discriminant validity across reflective constructs is detected [79].

**Table 2:** Measurement model results.

	Chi-square	CMIN/*df*	TLI	CFI	IFI	RMSEA
						
CFA model	794.159 (p < 0.001)	2.674	0.922	0.934	0.934	0.055
Aggregate model	924.902 (p < 0.001)	3.023	0.905	0.918	0.918	0.06
Multigroup model	1928.196 (p < 0.001)	1.575	0.90	0.912	0.914	0.032
Threshold	< 0.05	< 5	> 0.9	> 0.9	> 0.9	< 0.08
Assessment	Passed	passed	passed	passed	passed	passed

Note: TLI = Tucker–Lewis’s index; CFI = comparative fit index; IFI = incremental fit index; RMSEA = root mean square error approximation.

**Table 3:** Convergent validity and reliability.

Construct	Items	Factor loadings	Cronbach’s alpha	CR	AVE
Innovation Capability (IC)	IC1	0.722	0.847	0.841	0.571
	IC2	0.707			
	IC3	0.788			
	IC4	0.80			
Innovative Human Capital (IHC)	IHC1	0.764	0.799	0.803	0.576
	IHC2	0.745			
	IHC3	0.767			
Network Partnership Benefit (NPB)	NPB1	0.696	0.774	0.776	0.536
	NPB2	0.734			
	NPB3	0.767			
Strategic agility (SA)	SA1	0.748	0.844	0.844	0.575
	SA2	0.761			
	SA3	0.768			
	SA4	0.756			
Open Innovation Breadth and Depth (OBD)	OBD1	0.664	0.739	0.754	0.507
	OBD2	0.782			
	OBD3	0.685			
Collaboration-based Business Model Innovation (CBMI)	CBMI1	0.770	0.882	0.889	0.615
	CBMI2	0.759			
	CBMI3	0.798			
	CBMI4	0.798			
	CBMI5	0.796			
Open Innovation Cooperation (OIC)	OIC1	0.746	0.867	0.875	0.584
	OIC2	0.737			
	OIC3	0.803			
	OIC4	0.805			
	OIC5	0.726			

***Note***: AVE = average variance extracted; CR = composite reliability.

**Table 4:** Discriminant validity.

**Heterotrait-Monotrait Ratio of Correlations (HTMT)**							
	CBMI	OBD	OIC	SA	IC	IHC	NPB
**CBMI**							
**OBD**	0.696						
**OIC**	0.661	0.613					
**SA**	0.433	0.523	0.287				
**IC**	0.368	0.344	0.328	0.235			
**IHC**	0.301	0.392	0.429	0.180	0.554		
**NPB**	0.376	0.455	0.410	0.607	0.339	0.382	
**Fornell-Larcker Criterion**							
	**NPB**	**CBMI**	**IHC**	**OBD**	**IC**	**SA**	**OIC**
**NPB**	**0.784**						
**CBMI**	0.69	**0.712**					
**IHC**	0.648	0.606	**0.764**				
**OBD**	0.429	0.523	0.284	**0.758**			
**IC**	0.369	0.348	0.328	0.238	**0.755**		
**SA**	0.298	0.392	0.424	0.18	0.561	**0.759**	
**OIC**	0.373	0.455	0.405	0.607	0.343	0.382	**0.732**

### Step 2: Structural model

All structures are developed according to the model presented in Fig 1. The results of the GOF values satisfied the requirements provided by Hu and Bentler after evaluating the structural model [80]. CMIN/df (3.023), CFI (0.918), IFI (0.918), RMSEA. (0.06), and TLI (0.905) were acceptable fit (**Table 5**).

**Table 5:** Measurement invariance.

Fit index	Configural invariance (unconstrained model)	Metric invariance (equal factor loadings)	Scalar invariance (equal intercepts)	Threshold
CMIN/*df*	1.51	1.528	1.678	<3.00
CFI	0.924	0.918	0.91	>0.90
RMSEA	0.031	0.031	0.031	<0.08
Assessment	Acceptable	Acceptable	Acceptable	

### Step 3: Multigroup structural invariance

Measurement invariance analysis provides a guideline to determine if the measurement models in the two groups are statistically different [81]. It was feasible to determine whether respondents from the two groups understood the underlying questions in the identical questionnaires. According to the CFA model, the measurement invariance technique conducts the following additions: assignment constant generation, constant metric generation, and scalar constant generation. When the values are within a certain threshold, the load factor can compare the two groups. **Table 5** illustrates the continuous measurement invariance monitoring after the CFA model when the fit index is determined to pass the specified criteria.

## 5. Discussion and implications

The hypothesis testing was carried out using multigroup structural invariance to identify the open innovation strategy as an enabler between the resource-and-capability antecedents and family SMEs’ collaboration-based business innovation. This study also investigated how generational cohorts of interest think about collaboration-based business innovation.

This section shows how to look at aggregate-sample data, and **Table 6** shows the results of the structural model for a single group. We find that Thai family-owned SMEs tend to start attending their innovation capability and strategic agility. We could see that 0.1% significance level indicates the effects of innovative human capital (β = 0.273***) and strategic agility (β = 0.365***) on open innovation breadth and depth exist as confirmed by **H6** and **H8**. It is found that those SMEs are likely to look for network partnership benefits when they attempt to identify the number (breadth) and extent (depth) of external sources (β = 0.146*); this was supported by **H7**. Regarding the importance of innovation capability, no effect exists (β = 0.094; n.s.) when **H5** was not significantly supported. **H4** confirmed that open innovation breadth and depth significantly affect open innovation cooperation (β = 0.681***) at 0.1% significance level. It further implies that when family SMEs execute an open innovation strategy, they are more likely to first identify the number and extent of external knowledge providers they would partner with and then increase the partner breadth and depth until the cooperation plan is fulfilled. Open innovation cooperation and collaboration-based business innovation are linked (β = 0.68***), according to **H3**. This means that SMEs are more likely to start new businesses as a form of business model innovation when they have agreed on a cooperation strategy. Regarding an aggregate group of samples, the results reveal a good link between variables of interest. However, this study demonstrates the multigroup moderation analysis to gain more insights into how generational cohorts think about collaboration-based business innovation in the next section.

**Table 6:** Structural model.

H	Path relationships		Estimates	*p*	Results
H3	Open innovation breadth and depth	Open innovation cooperation	0.681	***	Supported
H4	Open innovation cooperation	Collaboration-based business model innovation	0.68	***	Supported
H5	Innovation capability	Open innovation breadth and depth	0.094	0.114	Rejected
H6	Innovative human capital	Open innovation breadth and depth	0.273	***	Supported
H7	Network membership benefit	Open innovation breadth and depth	0.146	*	Supported
H8	Strategic agility	Open innovation breadth and depth	0.365	***	Supported

Note:

* Significant at < .*05*, **Significant at < 0.*01*

***Significant at < 0.001.

Please refer to the multigroup structural model in **Table 7** and note that the generational cohort differences may become more noticeable in multigroup data, which may differ from aggregate data because strong group-specific findings might cancel each other out [82]. However, we return to the hypothesis testing of **H1** and **H2**, while **H3 –H8** are their subset assumptions that help answer both **H1** and **H2**. Regarding **H5**, our results show that innovation ability has no effect on the breadth and depth of open innovation among different generations of Thai family-owned SMEs. This may be because they still cannot sufficiently obtain new talents or resources to improve their firm’s innovation processes. They may perceive that their capabilities for R&D is still low and inefficient. So, they could not increase the number of new products and services introduced to the market. Like **H7**, there is no significant difference between network partnership benefits and open innovation breadth and depth. This may be because they still cannot see the clear benefits arising from network partnerships but continue seeking useful external knowledge partners. Our findings indicate that Gen-Y Millennials (β = 0.333**) and Baby Boomers (β = 0.313*) appear to pay their attention to the importance of innovative human capital as revealed in **H6**. When it comes to employment preferences and positive social change, Generation Y is quite similar to Baby Boomers in many ways. These two cohorts have values in common, they are able to collaborate with both internal and external partners. It is for this reason that they have placed a high value on human capital (and also social capital) [83]. To remain flexible in facing new developments, strategic direction, and innovative ways to create value [84], **H8** reveals that there are differences among zoomers, millennials, and boomers in determining strategic agility for open innovation breadth and depth. When comparing boomers with zoomers and millennials, our findings show that there are statistically significant differences. The critical ratio difference between millennials and boomers indicates a 15% loading difference and that millennials tend to be more agile than boomers. The 18.9% difference shows that zoomers tend to be more agile than boomers as well. But when comparing Zoomers to millennials, there appears to be no effect. We imply that when family business owners in the zoomer and millennial generations realize that they should respond to and capitalize on general/disruptive changes, provide effective IT-business support, and encourage quick internal adjustments whenever there is a shortage of resources, they are more likely to search for external knowledge sources needed to partner with while also dealing with consumer demands and deciding in the face of market changes. Finally, we found that 38.4% of the total variance in Generation Z, 54.4% of the total variance in Generation Y, 36.4% of the total variance in Generation X, and 19.5% of the total variance in Baby Boomer could be explained by the assumption that most small businesses are likely to agree with these four resource and capability antecedents when it comes to a partner-search strategy for new ideas and technologies. Hence, the confirmation of **H5 –H8** led us to the summary of **H2**, which is that most family SME owners in each generation tend to realize and act on the resource-and-capability antecedent factors differently. Based on our empirical evidence, we found that both millennials and boomers seemed to understand the value of innovative human capital when they were looking for, identifying, and choosing who would be good partners for open collaboration [30]. Looking at strategic agility, we found that Generation Z, Generation Y, and Baby Boomers were inclined to be more agile than Generation X when they understood that it was essential to making agile initiatives turn on (i.e., taking the time and support that was needed by internal stakeholders so they could change their behavior to match the new collaborative ones) before cooperation would be established.

**Table 7:** Multigroup structural model and critical ratio difference.

Hypothesized Relationship	Standardized loading				Critical ratio difference						Threshold
	Gen Z	Gen Y	Gen X	Gen B	X vs. Y	X vs. Z	X vs. B	Y vs. Z	Y vs. B	Z vs. B	
H4	0.376***	0.836***	0.977***	0.083	|0.904|	|-0.07|	|-7.165|*	|-0.876|	|-5.556|*	|-6.161|*	|1.96|
H3	0.518***	0.841***	0.853***	0.194	|0.596|	|3.838|*	|-6.544|*	|3.866|*	|-7.078|*	|-2.837|*	|1.96|
H5	0.047	0.114	0.288	0.132	|0.224|	|0.654|	|-3.391|*	|0.679|	|-6.017|*	|-4.78|*	|1.96|
H6	0.105	0.333**	0.085	0.313*	|0.428|	|0.557|	|0.46|	|0.214|	|0.921|	|0.996|	|1.96|
H7	0.173	0.046	0.239	-0.025	|1.769|	|-0.34|	|1.524|	|-2.165|*	|3.11|*	|1.236|	|1.96|
H8	0.499***	0.465**	0.057	0.31*	|1.211|	|0.322|	|-5.311|*	|-1.396|	|-8.717|*	|-8.847|*	|1.96|

Note: The critical ratio difference in absolute value corresponds to 1.96 at 5% significance level (p < 0.05*).

To execute open innovation strategy, our results show that there are differences among zoomers, millennials, and busters in determining breadth and depth for open innovation cooperation. They were supported by **H3**. Our model predicts that 14% of the fitted data in the Generation Z model, 69.8% of the fitted data in the Generation Y model, and 95.4% of the fitted data in the Generation X model could be explained by the fact that family business owners among these generations are more likely to increase both the partner-search breadth and depth and finally build the cooperation model with a necessary ingredient of triple/quadruple/quintuple helix stakeholders.

The evidence in support of **H4** shows that open innovation cooperation works together with collaboration-based business model innovation. Its effects on the development of collaboration-based business model innovation are estimated at 51.8% for zoomers, 84.1% for millennials, and 85.3% for busters. The results also indicate that when comparing zoomers to both millennials and busters, it appears that there are differences among them in terms of scaleups, demand aggregation insights, the value of collaboration stakeholders, and patterns of offering new solutions. All these are what zoomers, millennials, and busters think differently about collaboration-based business innovation. When we looked at **H3** and **H4** and found that they were true, we came up with the answer to **H1**, which is that family-owned businesses look at how many (external search breadth) and how deep (external search depth) their collaborations should be with a variety of different types of partners [29]. This strategy may imply a number and value of co-innovation investment projects, merger-and-acquisitions, and joint ventures, making business model innovation different depending on generation cohorts, especially zoomers, millennials, and busters. Overall, our data seemed to support the existence of multigroup moderation of generational cohorts.

### 5.1. Theoretical and practical implications

As a result of our present investigation, we feel that the findings are essential for both research and practice. In a theoretical perspective, this current research casts new light on the debate between the branch of literature hypothesizing that benefits from open innovation strategies are subject to collaboration-based business innovation. There are four key theorical implications proposed to the literature as follows.

1. First, this study presents the antecedent components of an open innovation approach that are based on resource and capability perspectives. The resource-and-capability antecedent factors of open innovation strategy are developed by incorporating innovation capability, innovative human capital, network partnership benefits, and strategic agility, all of which contribute to an emerging debate in the open innovation literature.
2. Second, open innovation strategy implementation includes both breadth and depth of strategic partner search, and cooperation with other open innovators. As the open innovation literature evolves to understand the concept of open innovation, such as Open Innovation 5.0, our findings show that open innovation as a cooperation plan should be taken into account by the innovation helix model to impact a broader range of stakeholders as new innovations are proposed to solve social issues.
3. Third, our current research provides a holistic approach to collaboration-based business model innovation. Our findings pay attention to the conceptualization of collaboration-based business model innovation which extends the insight into the open innovation literature. The findings show that there is an importance to business model innovation based on the collaborative network approach among generational cohorts of family-owned SMEs, especially Generation Z, Generation Y, and Generation X.
4. Fourth, we provide insights into the moderating role of generational cohorts by age in the business and innovation literature. Our findings contribute to the emerging literature which highlights the need for collaboration-based business model innovation as a family multigenerational-ownership SMEs in Thailand are more likely to work together, share resources, and use outside resources to come up with new solutions.

From a practical standpoint, our model gives us an insight of key resource-and-capability aspects of open innovation strategy and how these aspects relate to open innovation theory. However, our empirical evidence allows us to categorize our main findings into the following practical implications scenarios:

1. Our main findings reveal that innovative human capital and strategic agility appear to have the most powerful effect on the partner-search strategy of open innovation. This implies for SME practitioners that the value of human capital and the importance of strategic agility are necessary conditions for increasing business collaboration.
2. Among the respondents, three of four generations were associated with high tendencies for forming collaboration-based business model innovation. Therefore, business practitioners should be aware of their generational cohorts’ different shared value thoughts—especially zoomers, millennials, and busters—when coming to the formation of business model innovation through open innovation strategy.
3. Our findings suggest to business practitioners that they should implement open innovation, which begins with searching for, identifying, and selecting strategic partners. So, the breadth and depth strategy of open innovation will follow the general step of figuring out how SME collaboration actions will affect your stakeholders, which is a common step in the field of strategic management [85].
4. Moving to open innovation as a cooperation plan, our findings suggest that business practitioners should pay attention to the triple/quadruple/quintuple helix cooperation model. Our measures of open innovation cooperation further recommend the critical steps that SMEs should consider when they decide to cooperate with external knowledge partners. These steps include: (1) ensuring that innovation activities and projects are relevant and feasible; (2) consulting with local stakeholders to determine what should be proposed; (3) addressing funding, timing, and desired outcomes; (4) allocating resources to innovation activities and projects; and (5) executing innovation activities, projects, and risks. These key takeaways are sourced from **OIC 1–5**.

## 6. Conclusion

Considering the increasing value of open innovation economy [86], it is a relative new topic in the academic literature with limited empirical studies of open innovation strategy for collaboration-based business model innovation. The numerous studies have focused on Ahmad et al. (2020) [2], Greco et al. (2016) [29], Bengtsson et al. (2015) [30] and Zhu et al. (2019) [31], for instance, highlight the importance of openness in collaboration through external search breadth and depth and the business model innovation challenge in terms of novelty and efficiency in different countries’ context. However, the awareness of potential resource-and-capability antecedents of open innovation strategy is less concentrated when there is an argument about business model innovation based on network approach. The current research contributes to knowledge by identifying resource-and-capability antecedents of open innovation, dividing open innovation into partner-search strategy and cooperative strategy, and developing the measures of collaboration-based business model innovation. Our hypothesis testing and analysis provide key answers for our four central research questions. We found that innovative human capital, network partnership benefits, and strategic agility appear to be the antecedents of open innovation strategy execution in aggregate data. For more insight, we found that Generation Y and Baby Boomer consider innovative human capital and strategic agility as the most powerful antecedents of strategic open innovation breadth and depth. The importance of open innovation strategy in lights of partner breadth-and-depth and cooperation plan is relevant to the formation and tendency of collaboration-based family business model. These effects appear most in Generation Z, Generation Y, and Generation X.

Our empirical findings are subject to some limitations generally associated with structural survey-based research. First, our cross-sectional data collected during May–October 2021 is required to further investigate using a set of resource-and-capability antecedent variables. This is because innovation capability has not been statically confirmed; we will not conclude whether or not there is a role for innovation capability. The long-term study needs to make sure that innovation ability influences business model innovation. Second, research limitations exist in relation to the creation and development of open innovation breadth and depth, where our research design differed from the design suggested by Bengtsson et al. (2015) [30] in terms of matrix analysis of network partners. It would be better to use both measure designs to provide a better understanding of open innovation breadth and depth. This is due to the fact that our design cannot identify the *exact* number and extent of external partners who participate in the collaborative innovation activities and projects but provides relevant steps of open innovation breadth and depth.

## Appendix 1

**Table:** 

**Construct**	**Items**	**Measures**	**References**
Innovation capability	IC1	…constantly obtains new talents or resources to improve our firm’s innovation processes during the last five years.	Ahmad et al. (2020) [2] Liao et al. (2007) [65] Lin (2007) [66]
	IC2	…try out new ideas or ways to innovate within our sources during the last five years.	
	IC3	… have capabilities for R&D of new products or services at a certain level.	
	IC4	… increase the number of new products and services introduced to the market during the last five years.	
Innovative human capital	IHC1	… increase in the level of skills necessary to carry out the specific job.	McGuirk et al. (2015) [38] Chabbouh and Boujelbene (2020) [5] Vidotto et al. (2017) [67]
	IHC2	… encourage staff to join the training program during the last five years.	
	IHC3	… empower employees to be creative.	
Network partnership benefits	NPB1	… access to R&D partnerships and corporate collaborations	Chesbrough and Appleyard (2007) [68] Lee et al. (2010) [69] Del Vecchio et al. (2019) [1]
	NPB2	… solve business problems	
	NPB3	… create new revenue streams	
Strategic agility	SA1	… respond to and capitalize on general/disruptive changes	Cepeda and Arias-Pérez (2019) [70] Panda (2021) [71] Ashrafi et al. (2019) [72]
	SA2	… effective IT-business support	
	SA3	… encourage quick internal adjustments whenever there is a shortage of resources (manpower, funding, etc.)	
	SA4	… quick decision-making in the face of market changes	
Open innovation breadth and depth	OBD1	… search for external knowledge sources	Wang et al. (2020) [87]
	OBD2	… identify key stakeholders (such as suppliers, customers, universities, rivals, laboratory, local community etc.)	
	OBD3	… select key stakeholders to partner with	
Open innovation cooperation	OIC1	… Explore relevant and feasible activities and project	Kontinakis & Zhang (2018) [33] Kontinakis et al. (2019) [73]
	OIC2	… Consult local stakeholders to select what to Propose	
	OIC3	… Discuss their funding, timing and expected Results	
	OIC4	… Allocate probable resources	
	OIC5	… Execute and manage activities and projects (Including risks)	
Collaboration-based business model innovation	CBMI1	… scale up the collaboration-based business model	Zhu et al. (2019) [31] Kariv et al. (2014) [15]
	CBMI2	… enables demand aggregation	
	CBMI3	… incorporate transparency into the business model	
	CBMI4	… offer new solutions and combinations of products/services/knowledge	
	CBM5	… brings new collaboration stakeholders in the business model innovation	

## Supporting information

S1 Data (XLSX)
